# Total organic carbon, aluminium and iron in bulk samples and aggregate size fractions of a sandy clay loam humic soil under sugarcane relative to native forest in northern KwaZulu-Natal, South Africa

**DOI:** 10.1016/j.heliyon.2023.e14000

**Published:** 2023-02-26

**Authors:** Vusumuzi Erick Mbanjwa, Pardon Muchaonyerwa, Jeffrey Charles Hughes

**Affiliations:** a Soil Science Discipline, School of Agricultural, Earth and Environmental Sciences, University of KwaZulu-Natal, Private Bag X01, Scottsville 3209, South Africa

**Keywords:** Aggregate fraction, Humic soil, Sugarcane, Forest, Sandy clay loam

## Abstract

The distribution of total aluminium (Al) and iron (Fe) and organic carbon (TOC) in aggregate fractions gives an insight into the dynamics of these elements in soil. This study determined the effects of over 30 years of sugarcane cultivation, compared to adjacent native forest, on Al, Fe and TOC in bulk soil and aggregate fractions within the 100 cm depth of a sandy clay loam humic soil. Samples were separated into large macro-aggregates: LM (>2000 μm), small macro-aggregates: SM (250–2000 μm), micro-aggregates: M (250-63 μm) and silt + clay: SC (<63 μm) fractions. The TOC was analyzed by dry combustion and total Al and Fe by X-ray fluorescence spectrometry. Sugarcane cultivation (i) reduced macro-aggregates and TOC and (ii) increased the SC fraction and total Al and Fe. The mean weight diameter declined from 1.32 mm (0–30 cm) to 1.06 mm (30–100 cm) under forest. Average (0–100 cm) Al and Fe contents (g kg^−1^) increased in LM (6–16 for Al; 6 to 9 for Fe), SM (7–11 for Al), M (5–14 for Al; 6 to 9 for Fe) and SC (7–16 for Al; 9 to 10 for Fe) under sugarcane relative to forest. The TOC (g kg^−1^) declined in the LM (13–7) and SM (7–6) but increased in the M (5–9) and SC (10–13) due to cultivation. These findings suggested that sugarcane cultivation decreases aggregate stability and TOC in macro-aggregates, and increases Al and Fe in all aggregates. Adoption of practices inclined to improve or maintain TOC as well as liming to increase pH are necessary management practices for sustainable production.

## 1 Introduction

Aggregate stability (AS) is an important soil structural component [[1], [2], [3], [4]] as it controls the dynamics of soil organic matter (SOM) by protecting it within stable aggregates [3,4]. Numerous authors have discussed soil aggregate formation processes [1,[3], [4], [5], [6]]. Evidence from such studies suggests that AS is driven by (i) internal factors, such as clay mineralogy, organic matter, aluminium (Al) and iron (Fe) contents, and exchangeable cations, and (ii) external factors including climate, soil formation processes and land use and management.

The external factors alter the internal factors in a direct or indirect manner [2,7]. For example, some studies [[8], [9], [10], [11], [12], [13]] have shown that most of the negative effects on AS, following the conversion of natural ecosystems to arable agriculture, are largely a result of (i) a decreased supply of inputs due to management practices such as stubble burning, (ii) export of carbon (C) through the harvesting of plant matter, and (iii) higher rates of loss and reduction in soil organic carbon (SOC) with cultivation. Grohmann [14] reported that intensive cultivation of natural forest soils reduced the percentage of aggregates larger than 2 mm by about half in both Oxisols and Ultisols from Brazil. Tisdall and Oades [15] found that micro-aggregates (<0.25 mm) were less affected by cropping and management than macro-aggregates. Beare et al. [16] made a similar observation in a wide range of soils under different climates in the USA.

It is widely considered that the interaction of positively charged elements such as Fe and Al with clay or SOM can synergistically promote aggregation in soils, thereby improving structural stability through cationic bridging and formation of organo-mineral complexes [5,[17], [18], [19], [20], [21], [22], [23], [24]]. These materials may naturally be distributed unevenly in different size fractions of soil aggregates [15,25], and may be affected by a variety of land use management activities, including cultivation [8,21]. While the influence of land management practices on AS is well documented, much of the published research on the total Al, Fe and C distribution within different aggregate size classes has focused on the top 30 cm of soil profiles [17,26,27].

The organic matter in surface layers is affected by tillage operations and processes of addition and decomposition due to the availability of oxygen [20,28,29]. In deeper layers a lower supply of oxygen may limit decomposition [24,[30], [31], [32]]. The decline in total C with depth provides an opportunity to study the relationships between C, Al and Fe and their contribution to aggregation. These relationships and effects of management are not clearly understood for humic soils, which are highly weathered and leached, and have high organic C (>1.8%) in the topsoil and extremely low base status (<4 cmol_c_ of exchangeable bases per kg clay for every one percent SOC present) [33]. The clay mineralogy of humic soils typically consists of kaolinite, aluminous chlorite, gibbsite and iron oxides (goethite and hematite). The humic soils are, however, physically very stable due to their strong micro-aggregation [34] that promotes balanced porosity against various stresses such as the impact of raindrops, erosive forces and contraction and swelling caused by drying and rewetting [2,35]. Many humic soils have been converted to various agricultural uses and the effects of such changes on AS have not been well studied.

The primary objectives of this study were, thus, to determine the effects of land use change from native forest to sugarcane farming on (i) AS and size distribution, and (ii) the distribution of total Al, Fe and C within different aggregate size fractions to a 1 m depth in some humic soils. The 1 m sampling depth was employed to avoid the underestimation of the SOC and related properties especially in the subsoil as studies have found high proportions of SOC (from 46 to 63%) in the horizons below 30 cm [e.g. 24, 28, 29, 30, 31, 32]. This study is important for the management of humic soils, as the results will indicate the long-term structural effects of putting highly weathered, acid soils with high SOC under intensive agricultural production.

## 2 Materials and methods

### 2.1 Site description

The study was conducted near Eshowe (28^o^ 52.763′ S; 31^o^ 25.180′ E) in northern KwaZulu-Natal, South Africa. The mean annual rainfall is 1109 mm and mean monthly temperatures range from 29.1 °C in January to 11.3 °C in June. The site is located on a relatively flat landscape (0–2% slope) at an average altitude of 550 m a.s.l. The area under commercial sugarcane (*Saccharum officinarum*) had undergone pre-harvest burning for more than 30 years [36]. The sugarcane is not irrigated and is commonly fertilized with 130 kg N ha^−1^, 20 kg P ha^−1^ and 140 kg K ha^−1^ as 5:1:5 (46) at 650 kg ha^−1^ approximately 45 days after harvesting [37]. Dolomitic lime is also applied (1–10 t ha^−1^) to reduce acid saturation levels to 20% at least once every 10 years. Sunn hemp (*Crotalaria juncea*) or oats (*Avena sativa*) are usually planted as rotation crops before replanting sugarcane [38]. The indigenous coastal scarp forest (native forest) was approximately 50 m from the sugarcane field and was used as the control as it had received no fertilizer, lime or irrigation. The soil type under both land uses was Magwa 2200 [33]; Xanthic Ferralsol [38] formed on Natal Group sandstone (Eshowe member) [39]. The Eshowe member generally consists of 85–95% coarse to very coarse-grained, immature, poorly-sorted sandstone with subordinate interbedded reddish micaceous shales, siltstones and unweathered feldspar [40].

### 2.2 Soil sampling

Soil samples were collected from three subplots of approximately 0.1–0.3 ha per land use that were at least 20 m apart. Three replicate bulk soil samples were collected from the face of a 1.2 m deep profile pit at depth intervals of 0–5, 5–10, 10–15, 15–20, 20–30, 30–40, 40–50, 50–60, 60–80 and 80–100 cm giving a total of 90 samples from each land use type. All the samples were air-dried, and about one third of each sample was used for AS measurements, while the remainder was ground with a pestle and mortar and passed through a 2 mm sieve. Malepfane et al. [41] found that the SOC ranged from 30 to 72 g kg^−1^ in the top 30 cm and 18–38 g kg^−1^ below 30 cm on the same soils. Yost and Hartemink [42] suggested that SOC concentrations are highest in the top 30 cm and gradually decrease with depth, and therefore the lower limit of the topsoil was taken to be at 30 cm with material below this point (30–100 cm) termed subsoil in this study.

### 2.3 pH, particle size distribution, colour and clay mineralogy

Soil pH was determined with a Metrohm E396B meter in a 1:2.5 soil: 1 M KCl suspension using a glass electrode. Particle size distribution was determined using the hydrometer method [43]. Soil colour was described using a Munsell Colour Chart. Clay mineralogy was determined by X-ray diffraction (XRD) on Ca-saturated, oriented samples using a Bruker D8 ADVANCE diffractometer equipped with a Lynx Eye detector with Ni-filtered Cu-Kα radiation (λ = 0.154 nm) at 40 kV and 40 mA. The air-dried, glycerolated and heated (500 °C for 3 h) clay samples were scanned from 2° to 15° 2θ with a scanning step size of 0.01313° at 0.779 s per step [44].

### 2.4 Aggregate stability

The samples used for AS were sieved to collect sufficient aggregates between 2.8 and 5.0 mm. Aggregate size separation was carried out using the method adapted from Elliott [45]. The method involved separation of aggregates by wet sieving the air-dried soil through a series of three sieves to isolate four aggregate size classes, which are referred to here as i) large macro-aggregates: LM (>2000 μm), ii) small macro-aggregates: SM (250–2000 μm), iii) micro-aggregates: M (63–250 μm), and iv) silt + clay: SC (<63 μm). A 100 g sub-sample was evenly spread on top of the 2000 μm sieve, submerged in deionized water at room temperature for 5 min, resulting in slaking of the soil, which was subsequently sieved to separate water-stable aggregates by moving the sieve up and down 50 times over a period of 2 min. The material remaining on the 2000 μm sieve, i.e. the LM fraction, was back-washed into a beaker for drying. Soil plus water that passed through the sieve was poured onto the 250 μm sieve and the sieving procedure repeated. This was repeated for the 63 μm sieve. All aggregate classes were oven dried at 40 °C (48 h), weighed and stored at room temperature for the analysis of total Al, Fe and C. Mean weight diameter (MWD) was calculated using Equation (1):

Equation 1 $$MWD=\sum_{i=1}^{n}x_{i}w_{i}$$

where: $x_{i}$ is the mean diameter (mm) of any particular size range of aggregates separated by sieving, $w_{i}$ is the weight fraction of aggregates remaining on the sieve (%), and *n* is the number of aggregate classes separated.

### 2.5 Total aluminium, iron and carbon

Total Al and Fe were determined in both the bulk samples and the soil aggregates. Samples of each of the three largest aggregate size fractions were manually ground with a pestle and mortar to pass a 63 μm sieve. Each soil sample (2 g), from the different fractions, was mixed with 1 g of cellulose flakes as a binder, compressed into a pellet (19 mm diameter) under a pressure of 2 t cm^−2^, and analyzed for total Al and Fe using a polarized energy dispersive X-ray fluorescence spectrometer (X-LAB 2000 PED-XRF) with rhodium as the excitation source.

Total C values of the bulk soil samples from Malepfane et al. [41], for the same site and land uses, were used in this study while the total C within the aggregate size fractions was determined on finely ground samples (<0.5 mm) using a LECO CNS 2000 analyser. Since these soils were acidic and no carbonates were detected, the total C measured was considered to represent organic C and is henceforth referred to as total organic C (TOC).

### 2.6 Statistical analysis

Data were analyzed with Genstat 18 [46] by a two-way analysis of variance (p ≤ 0.05) to test the effects of (i) land use, soil depth, and their interaction for bulk soils, and (ii) land use, aggregate size fraction, and their interaction for individual depths. Differences between the means of the significant factor were assessed with Duncan's multiple range test (p ≤ 0.05). Least significant differences (LSD) at p = 0.05 were computed to separate treatment means for all properties. Linear regression analyses between TOC and total Al or Fe were also performed separately for forest soils and those under sugarcane, and all results were based on three replications in the field.

## 3 Results

### 3.1 Properties of the forest and sugarcane soils

The topsoil (0–30 cm) of the studied sites was a very dark brown (10 YR 2/1 to 2/2), fine, subangular blocky humic A horizon, and the subsoil (30–100 cm) was dark yellowish brown (5 YR to 7.5 YR 4/6) with apedal to weak structure. The clay mineralogy of the soils under both land uses was dominated by kaolinite, with subsidiary interlayered chlorite, goethite and quartz. The pH ranged from 4.03 to 4.75 and from 4.08 to 4.52 under forest and sugarcane, respectively (Table 1). There were no significant differences in pH between land uses except that soil under forest had a higher pH in the 0–5 cm depth, and lower in the 60–80 and 80–100 cm depths, than under sugarcane (Table 1).

**Table 1:** pH and particle size distribution of humic soil profiles under native forest and sugarcane (n = 3).

Land use	Depth (cm)	pH (KCl)	Clay Silt Sand (<0.002 mm) (0.002–0.05 mm) (0.05–2 mm) (%)		
Forest	0–5	4.75^ga^^a^	16.5^ghi^	19.2^hjk^	64.3^b^
Sugarcane		4.08^ab^	16.5^ghi^	3.2^a^	80.3^h^
Forest	5–10	4.18^abcd^	12.5^cde^	24.6^l^	62.9^b^
Sugarcane		4.09^ab^	16.5^ghi^	2.5^a^	80.9^h^
Forest	10–15	4.05^ab^	16.5^ghi^	20.5^hjk^	63.0^b^
Sugarcane		4.11^abc^	15.8^fgh^	9.9^bc^	74.3^fg^
Forest	15–20	4.03^a^	11.2^abc^	16.5^efghi^	72.3^defg^
Sugarcane		4.18^abcd^	16.6^ghi^	8.5^b^	74.9^g^
Forest	20–30	4.14^abc^	14.4^efg^	11.8^bcd^	73.8^efg^
Sugarcane		4.25^cde^	14.6^efg^	12.5^cde^	72.9^efg^
Forest	30–40	4.13^abc^	11.9^bcd^	17.2^fghij^	70.9^cde^
Sugarcane		4.16^abc^	15.2^fg^	13.2^cde^	71.6^cdef^
Forest	40–50	4.13^abc^	13.8^def^	14.5^def^	71.7^cdef^
Sugarcane		4.19^bcd^	18.5^ij^	12.6^cde^	68.9^c^
Forest	50–60	4.14^abc^	9.9^ab^	16.5^efgh^	73.6^efg^
Sugarcane		4.08^ab^	17.8^hij^	13.1^cde^	69.1^c^
Forest	60–80	4.14^abc^	11.9^bcd^	15.8^efg^	72.3^defg^
Sugarcane		4.34^e^	19.9^j^	10.5^bcd^	69.6^cd^
Forest	80–100	4.31^de^	9.2^a^	18.5^ghij^	72.3^defg^
Sugarcane		4.52^f^	17.9^hij^	22.5^kl^	59.6^a^

a Means within the same column followed by different letters are significantly different (p ≤ 0.05).

There were no significant differences in sand content between land uses, except that in soil under sugarcane it was significantly higher in the 0–15 cm depth and lower at 50–60 and 80–100 cm than under forest (Table 1). The silt content under forest was significantly higher at 0–20, 30–40 and 60–80 cm but was lower at 80–100 cm than sugarcane, while no significant differences were observed at other depths (Table 1). The clay content was significantly higher under sugarcane than under forest, except at 0–5, 10–15 and 20–30 cm, where there were no differences. The topsoil (0–30 cm) averaged 67 and 77% sand, 19 and 7% silt, and 14 and 16% clay under forest and sugarcane, respectively. The subsoil under forest and sugarcane had average values of 72 and 68% sand, 17 and 14% silt, and 11 and 18% clay, respectively. The overall (0–100 cm) textural class of the soils was sandy clay loam.

**Fig. 1:**
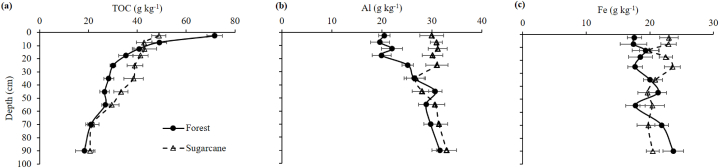
The concentration (±standard error) of (a) total organic carbon (TOC), (b) total aluminium (Al) and (c) total iron (Fe) with depth (0–100 cm) in bulk soil under native forest and sugarcane (n = 3).

The TOC was significantly lower in the top 5 cm and higher at 20–40 cm depth under sugarcane than under forest (p ≤ 0.05), with no significant differences at other depths between land uses (Fig. 1a). The TOC concentration significantly decreased with depth (p ≤ 0.05) under both land uses and the overall (0–100 cm) TOC was not significantly different between the land uses (p ≥ 0.05).

Total Al was significantly higher under sugarcane than under forest (p ≤ 0.05) in the top 30 cm, with no differences in deeper layers (Fig. 1b). The concentration of total Al increased with depth under forest but not under sugarcane. Similarly, total Fe was significantly greater (p ≤ 0.05) under sugarcane than under forest in the top 30 cm, except at 10–15 cm (Fig. 1c). Below 30 cm, the total Fe was not significantly different between land uses except in the 80–100 cm layer where it was lower under sugarcane.

### 3.2 Soil aggregate stability and size distribution

The distribution of the aggregate size fractions differed depending on land use and depth (p ≤ 0.05; Table 2). The proportion of LM was higher under forest in the top 15 cm, with no significant differences between land uses at other depths. The LM proportion did not change with depth under sugarcane, while it decreased under forest. On the other hand, the proportions of SM and M under forest were not significantly different to those under sugarcane except in the top 10 cm where these fractions were lower under forest. The SM proportion did not change with depth under sugarcane, while it increased under forest. The SM generally made up a higher proportion of the total than the LM at all depths except in the top 15 cm of the forest soil (Table 2). The proportion of M was generally lower than that of the SM, while the SC fraction had the lowest proportion irrespective of land use and soil depth. The proportion of SC was higher under sugarcane than under forest in the top 20 cm, with no differences in deeper layers and did not change with depth under sugarcane, whereas it increased under forest. There were no significant differences in the MWD between forest and sugarcane soils at all depths, except at 0–10 cm, where the forest soil had higher values. The MWD did not change with depth under sugarcane, while it decreased under forest from an average of 1.32 in the topsoil to 1.06 mm in the 30–100 cm depth (Table 2).

**Table 2:** Distribution of water stable aggregates and the mean weight diameter (MWD) of humic soil profiles under native forest and sugarcane (n = 3).

Land use	Depth (cm)	Aggregate size distribution (Mass %)				MWD (mm)
		>2000 μm	250–2000 μm	63–250 μm	<63 μm	
Forest	0–5	65.7^e^^a^	19.8^a^	11.8^abc^	2.7^a^	1.5^d^
Sugarcane		28.8^abc^	45.0^cdefg^	20.2^abcdef^	5.9^cdef^	1.2^abc^
Forest	5–10	58.4^e^	29.4^ab^	8.4^a^	3.8^ab^	1.5^d^
Sugarcane		16.1^abc^	48.0^defgh^	25.6^def^	10.3^g^	1.0^ab^
Forest	10–15	53.4^de^	33.6^bc^	8.7^ab^	4.3^abc^	1.4^cd^
Sugarcane		30.8^abc^	42.9^cde^	16.9^abcde^	9.4^ef^	1.2^abc^
Forest	15–20	31.8^bc^	46.7^defgh^	15.0^abcd^	6.5^abcd^	1.1^ab^
Sugarcane		13.6^abc^	54.3^efgh^	24.4^def^	7.7^efg^	1.0^a^
Forest	20–30	23.7^abc^	48.5^defgh^	22.2^bcdef^	5.6^bcde^	1.1^ab^
Sugarcane		25.1^abc^	48.1^defgh^	19.7^abcdef^	7.1^cdef^	1.1^ab^
Forest	30–40	11.6^ab^	57.5^h^	25.3^def^	5.6^cdef^	1.0^a^
Sugarcane		23.1^abc^	49.1^efgh^	19.4^abcdef^	8.4^fg^	1.2^abc^
Forest	40–50	7.1^a^	55.9^fgh^	29.1^f^	7.9^efg^	0.9^a^
Sugarcane		27.9^abc^	47.3^defgh^	19.3^abcdef^	5.5^abcde^	1.3^abc^
Forest	50–60	11.3^ab^	56.7^gh^	26.3^ef^	5.7^abcde^	1.0^a^
Sugarcane		29.4^bc^	42.9^cdef^	21.2^bcdef^	6.5^abcde^	1.2^abc^
Forest	60–80	22.5^abc^	52.6^efgh^	21.0^bcdef^	3.9^abcd^	1.1^ab^
Sugarcane		29.3^bc^	42.9^cde^	22.9^cdef^	4.9^abcde^	1.2^abc^
Forest	80–100	31.0^bc^	42.9^cdef^	19.4^abcdef^	6.7^def^	1.3^abcd^
Sugarcane		29.4^bc^	50.3^efgh^	14.9^abcde^	5.4^abcde^	1.4^bcd^

a Means within the same column followed by different letters are significantly different (p ≤ 0.05).

### 3.3 Total organic carbon in the aggregate size fractions

**Fig. 2:**
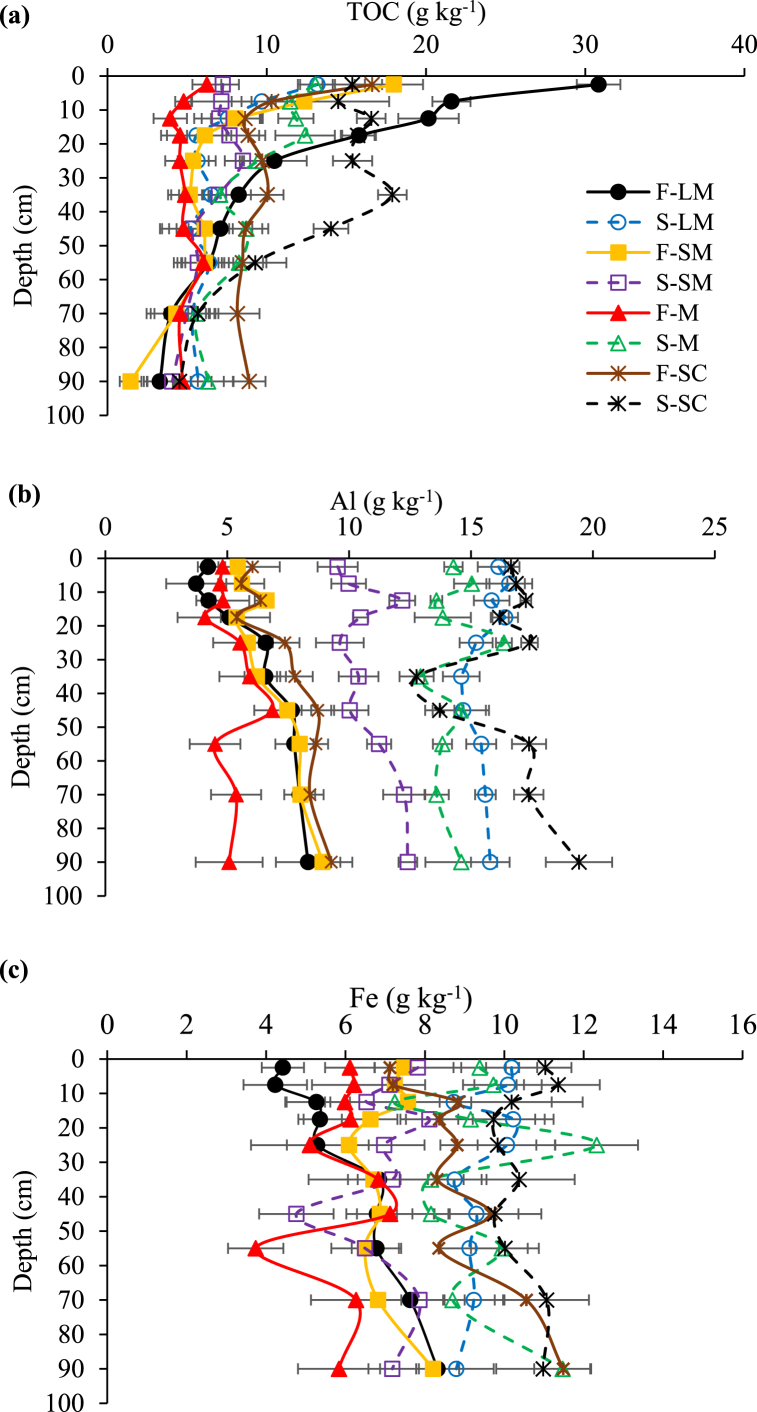
The concentration (±standard error) of (a) total carbon (TOC), (b) total aluminium (Al) and (c) total iron (Fe) with depth in large macro-aggregates (LM), small macro-aggregates (SM), micro-aggregates (M) and the silt + clay fraction (SC) of humic soils under native forest (F) and sugarcane (S) (n = 3).

The distribution of TOC within the aggregate size fractions differed depending on land use and aggregate size fraction (p ≤ 0.05; Fig. 2a). The TOC concentration in the LM and SM fractions in the top 15 cm was lower under sugarcane than forest. The TOC contents of the SM and M at 40–50 cm and the SC fraction at 30–50 cm were higher under sugarcane compared to forest (Fig. 2a). The greatest decline of TOC concentration with depth occurred in the top 15 cm below which no consistent TOC trend was observed in most aggregate size fractions under sugarcane. Within the total profile depth (0–100 cm), the TOC in the LM (13 and 7 g C kg^−1^) and SM (7 and 6 g C kg^−1^) fractions was 85 and 17% higher under forest than sugarcane, respectively. On the other hand, the TOC in the M (9 and 5 g C kg^−1^) and SC (13 and 10 g C kg^−1^) fractions was 80 and 13% higher under sugarcane than forest, respectively. The TOC in aggregates under forest was generally higher at 0–15 cm and lower at 20–50 cm when compared to sugarcane, with the exception of the SC fraction.

### 3.4 Total aluminium and iron in the aggregate size fractions

At all depths the Al concentration was significantly higher (p ≤ 0.05) in all aggregate size fractions under sugarcane compared to forest (Fig. 2b). The average (0–100 cm) Al content was 167, 57, 180, and 129% greater under sugarcane than forest in the LM (16 and 6 g Al kg^−1^), SM (11 and 7 g Al kg^−1^), M (14 and 5 g Al kg^−1^) and SC (16 and 7 g Al kg^−1^), respectively.

In the top 10 cm, total Fe concentration in the LM, M and SC fractions was significantly higher (p ≤ 0.05) under sugarcane compared to forest with no differences in the SM fraction (Fig. 2c). At 20–30, 50–60 and 60–80 cm, the Fe concentration was higher in the LM and M under sugarcane than forest with no differences in the SM and SC fractions. At 80–100 cm, total Fe was higher in the M fraction under sugarcane (12 g Fe kg^−1^) than forest (6 g Fe kg^−1^) with no significant differences in the other size fractions. The average (0–100 cm) Fe content was 50% higher under sugarcane than forest in the LM and M (9 and 6 g Fe kg^−1^), and 11% higher in the SC (10 and 9 g Fe kg^−1^) fractions.

### 3.5 Relationship between carbon, aluminium and iron in bulk soils and aggregate size fractions

**Fig. 3:**
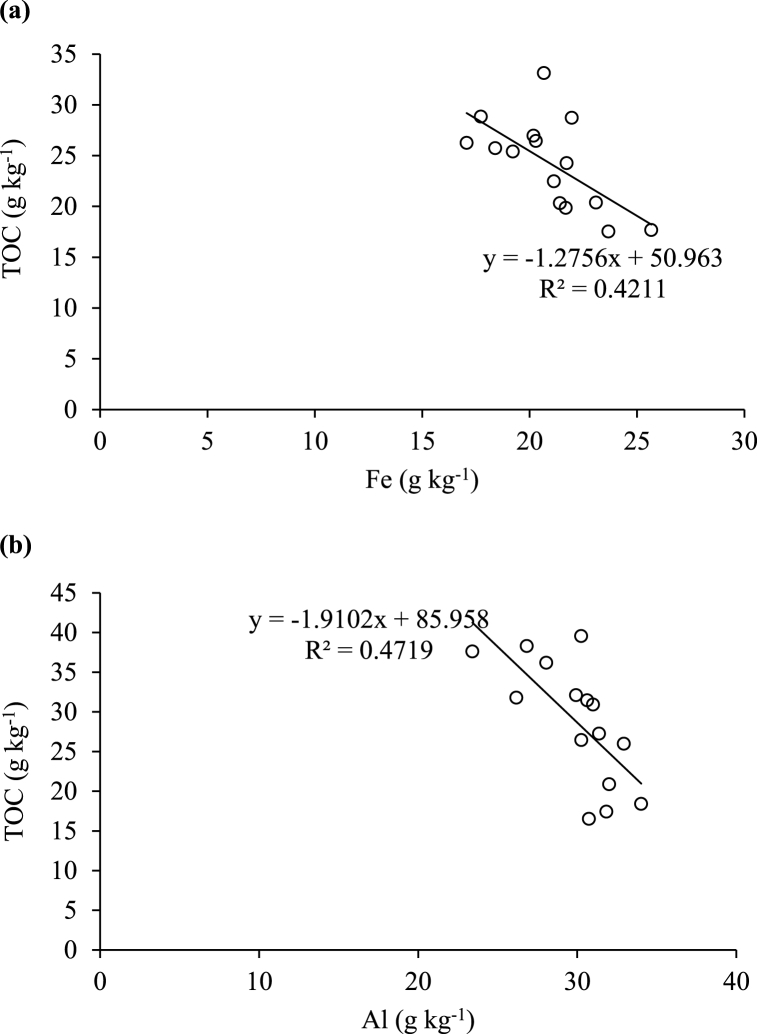
Relationship between total organic carbon (TOC) and (a) total iron (Fe) under native forest and (b) total aluminium (Al) under sugarcane in the humic subsoils (30–100 cm).

**Fig. 4:**
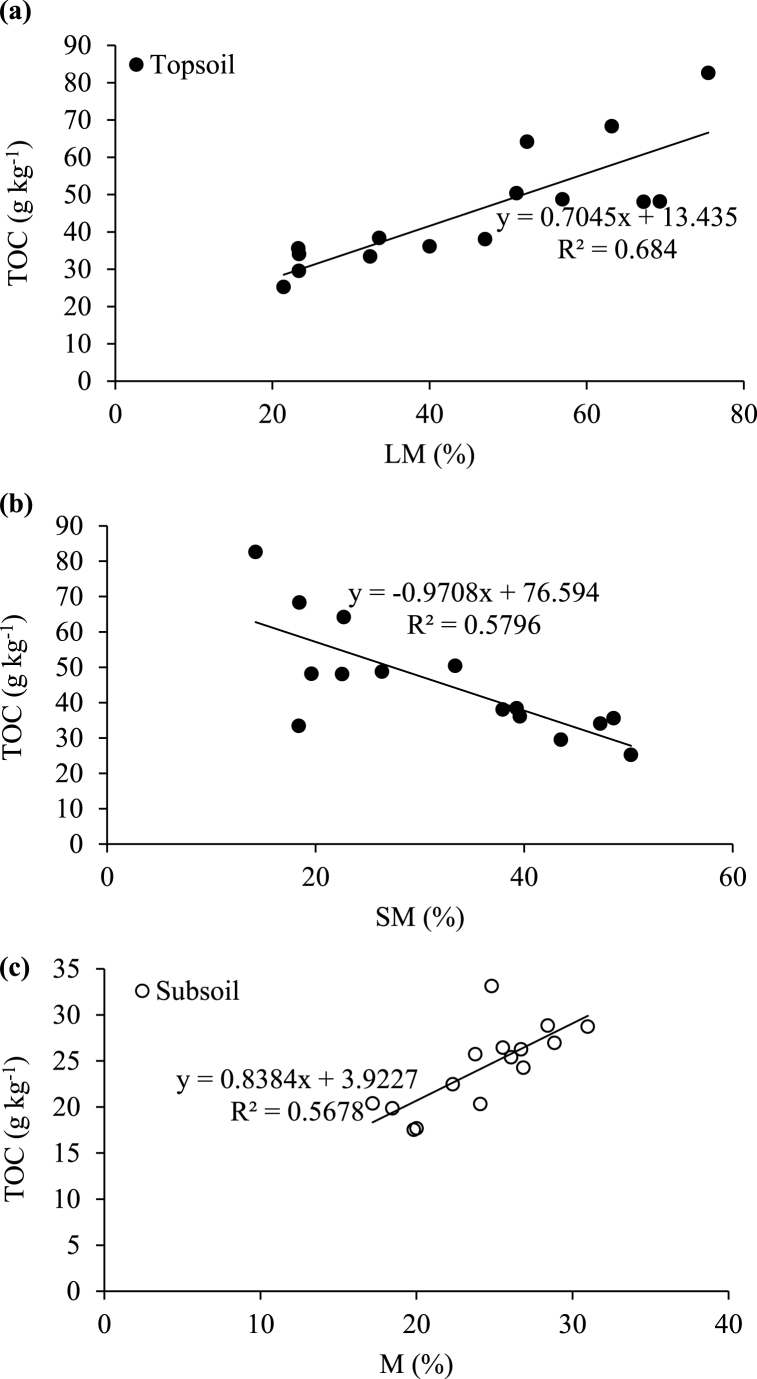
Relationship between total organic carbon (TOC) and (a) large macro-aggregates (LM), (b) small macro-aggregates (SM) and (c) micro-aggregates (M) in the humic topsoils (0–30 cm) and subsoils (30–100 cm) under native forest.

There were few significant relationships between the measured variables and only the most significant are discussed. Under forest, TOC in bulk soils was positively correlated with the proportion of LM in the topsoil (R^2^ = 0.68; Fig. 4a) but negatively with the SM fraction (R^2^ = 0.58; Fig. 4b). In the subsoil, the TOC was positively correlated with the M fraction (R^2^ = 0.57; Fig. 4c). No significant relationships were observed between the proportion of aggregate size fractions and TOC in the soils under sugarcane. There were also no significant relationships between total Al or Fe and the proportions of any of the aggregate size fractions under both land uses. The relationships of TOC to Fe under forest (R^2^ = 0.42; Fig. 3a) and Al under sugarcane (R^2^ = 0.47; Fig. 3b) in bulk subsoils were negative.

**Fig. 5:**
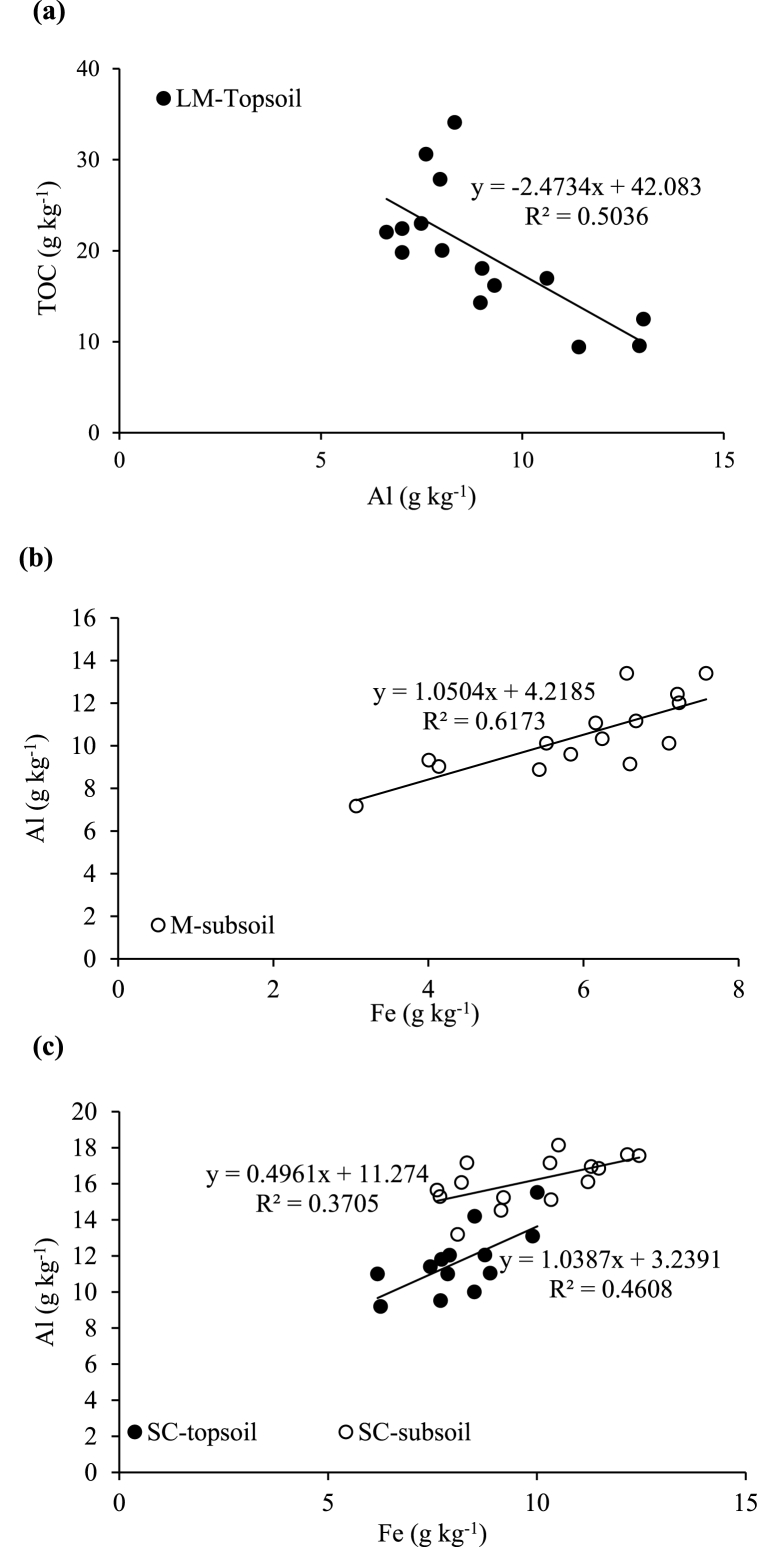
Relationship between (a) total organic carbon (TOC) and total aluminium (Al) in large macro-aggregates (LM), (b) total Al and total iron (Fe) in micro-aggregates (M) and (c) total Al and total Fe in the silt + clay (SC) fraction in the humic topsoils (0–30 cm) and subsoils (30–100 cm) under native forest.

**Fig. 6:**
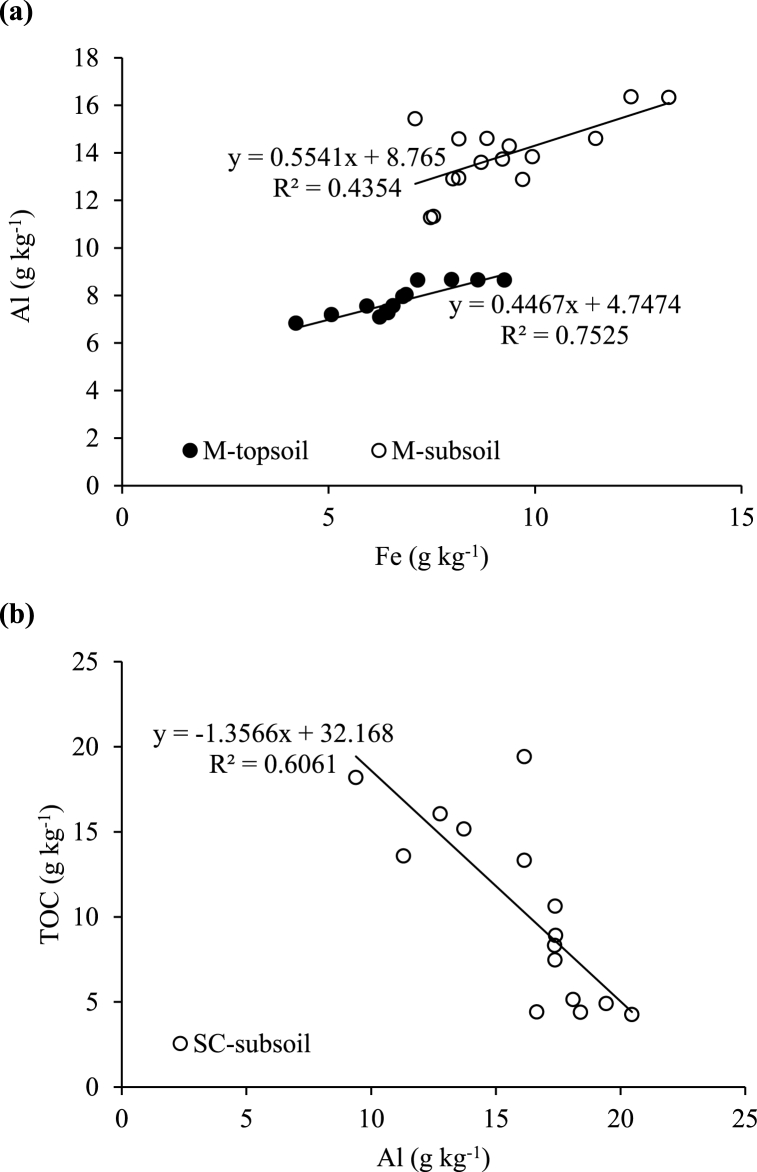
Relationship between (a) total aluminium (Al) and total iron (Fe) in micro-aggregates (M) of the humic topsoils (0–30 cm) and subsoils (30–100 cm), and (b) total organic carbon (TOC) and total Al in the silt + clay (SC) fraction of the subsoils under sugarcane.

Under forest, a negative relationship was observed between TOC and Al in the LM fraction of the topsoil (R^2^ = 0.50; Fig. 5a). A stronger relationship was observed between TOC and Fe in the M fraction of the subsoil (R^2^ = 0.62; Fig. 5b). The relationships between these variables were also positive but weaker for both the topsoil (R^2^ = 0.37) and subsoil (R^2^ = 0.46) in the SC fraction under forest (Fig. 5c). Under sugarcane, the relationship between Al and Fe was strong in the M fraction of the topsoil (R^2^ = 0.75) but weaker for the subsoil (R^2^ = 0.44) (Fig. 6a). The relationship between TOC and Al in the SC fraction of the subsoil was strong and negative (R^2^ = 0.61; Fig. 6b).

## 4 Discussion

### 4.1 Properties of the bulk soils

The lower pH in the 0–5 cm depth of the sugarcane soils could be attributed to the regular addition of acidifying fertilizers [6,19,47,48]. In most cropping practices, soil acidification problems are related to (i) the use of ammoniacal fertilizers, which encourage the displacement of basic cations by NH_4_^+^, (ii) nitrification (2 mol of H^+^ is produced per mole of NH_4_^+^) and (iii) toxicity of Al and other metals [[48], [49], [50]]. The higher total Al in the soils under sugarcane suggests that its hydrolysis in these acidic soils could further lower soil pH [48]. Although the soils under sugarcane are also limed, the application of 1–10 t lime ha^−1^ once every 10 years may not be sufficient to neutralize the acidity produced by annual fertilization and nitrification, together with potential hydrolysis of Al. Texturally, the soils from both land use types were similar (sandy clay loams). In the topsoils, the average clay content under forest (13%) was slightly lower than that under sugarcane (16%) and both were lower than those reported by Gubevu [51] for shallow (<30 cm) humic soils in the Ngome forest (30%) and sugarcane (42%) plantations in KwaZulu-Natal. This difference may be because the soils in the Gubevu [51] study were derived from dolerite under higher rainfall (>1500 mm p. a.) and higher altitude (1300 m a.s.l) conditions compared to those of the present study area. The predominance of sand at both land uses was inherited from the sandstone parent material, while the highly weathered state of the soils was indicated by the dominance of kaolinite in the clay fraction.

The TOC in the bulk soils supports the large number of studies that have found strong evidence for a decline of 30–80% in C content when forests are converted to arable agriculture [8,10,19,36,48,[52], [53], [54], [55]]. In all these studies, the loss of C has often been attributed to (i) erosion, (ii) lower C inputs, (iii) a reduced stabilisation of SOM due to reduced aggregation, and (iv) subsequent mineralization promoted by increased soil temperature and aeration. As expected the TOC decreased from the topsoil to subsoil layers due to the continuous aboveground C input by vegetation residues [30,31] and the lack of soil disturbance more especially under native ecosystems [11,25,48]. The higher TOC under sugarcane than forest in the 20–40 cm depth may possibly be a result of the translocation of C to lower depths [19,30,31,48,56] and contribution of root biomass [4].

The higher Al and Fe contents in the bulk sugarcane topsoils compared to those under forest could possibly be a result of the dilution by high organic matter in the forest soil, as SOC was not removed before the analysis of Al and Fe in these soils. Using such samples would have masked the concentration of Al and Fe in the soil that would otherwise be observed if the SOC was removed prior to the analysis of these elements [51,57,58]. Compared to forest (6 g Fe kg^−1^) soil, Yost et al. [59] found four times higher total Fe content in a bulk sandy loam soil (0–100 cm) under agriculture (23 g Fe kg^−1^) in the Central Sand Plains of Wisconsin. Other researchers [e.g. 36, 47] have reported an increase in the total Fe and Al content of tropical soils following application of N and P inorganic fertilizers at planting. In both these studies, Al was found to be chemically stabilized by interaction with organic matter such that the reduction in organic matter following cultivation increased the concentration of this element in the soil. The notable increase in Fe and Al content in the subsoils again corresponds with lower TOC, thus lower dilution [51,58].

### 4.2 Soil aggregate stability and size distribution and organic carbon in aggregate fractions

The higher MWD in the forest soil surface layers may possibly be a result of the minimal soil disturbance and higher TOC, thus favouring the formation of more stable aggregates [53,60]. Blair [61] also reported a significant reduction in the MWD of sugarcane soils compared to undisturbed grasslands in Australia.

The lower MWD values measured in the subsurface layers of the forest soils may not only be a result of lower TOC but could also be due to the lower clay (11%) content than in the sugarcane subsoil (18%) [4,62]. Aggregate stability has generally been found to increase with increasing clay content [6,11,19] especially in soils with non-expanding, crystalline clays, such as kaolinite, that are less dispersive [63]. Denef and Six [21] suggested that clay minerals may interact with organic matter through the formation of organo-mineral assemblages which, in turn, affect aggregation. The lack of significant differences in the MWD between the forest and sugarcane soils (p = 0.325) was perhaps, at least in part, because both land uses are quite undisturbed and so inputs of organic matter and decomposition rates may be similar under both land use systems since soils under sugarcane are only disturbed at replanting which occurs about every eight years at the locality.

The higher proportion of the LM in the surface layers of the forest soils than under sugarcane could be attributed to higher SOC, which could include live and decaying plant roots, fungal hyphae, and casts of earthworms and termites, which are rapidly destroyed by cultivation [4,62]. This finding is similar to that of Roth et al. [64] who reported a higher proportion of LM in the surface layer (0–10 cm) of virgin forest soils compared to sugarcane for a similar soil type at Londrina, Brazil. The substantial loss of TOC in the LM and SM fractions of the sugarcane soils was expected as the break-up of macro-aggregates and increased aeration caused by ploughing both favour decomposition of SOM [16,65], thereby reducing the TOC concentration [3,56,66]. The addition of mineral fertilizers and lime to the soils under sugarcane could have increased microbial activity to the extent that SOM was decomposed, lowering TOC. Castro Filho et al. [67] found the TOC content to be three times greater in macro-aggregates under forest (39 g C kg^−1^) compared to sugarcane (13 g C kg^−1^) in a Rhodic Ferralsol from southern Brazil. Based on a meta-analysis using data from 74 publications from around the world, Guo and Gifford [52] reported a 42% average loss of the antecedent TOC pool from native ecosystems on conversion to croplands although the authors did not indicate whether sugarcane was among the crops investigated.

With the exception of the Fe in the SM fraction, the higher total Al and Fe in all aggregate size fractions under sugarcane than in the forest soils, suggested that both are involved at various levels in the aggregation hierarchy of these soils, and are affected by changes in land use and soil management [2,26,68]. The decrease in the proportion of LM with depth under forest may be due to the lower SOM content at greater depth [30,60,64,65]. On the other hand, the higher proportion of SM in the surface layers of sugarcane soils than under forest suggests that tillage and cropping result in the mineralization of the organic C from larger aggregates causing the breakdown of LM to SM [53,61].

The effects of different land use and management were less pronounced for the M and SC fraction, possibly due to similarities in TOC, Al and Fe contents [14,52,58]. These results are, however, in contrast with the findings of Zhang et al. [69], who reported an increase of the M and SC fractions 20 years after native grassland was converted to maize farming in China. These contradictory findings could be associated with the crop species. Sugarcane fields are not ploughed as often as maize fields, which generally results in aggregates that are less resistant to change compared to the more frequently ploughed maize soils [48,56,67].

### 4.3 Relationship between carbon, aluminium and iron in bulk soils and within aggregate size fractions

The negative relationship observed between TOC and Fe (R^2^ = 0.42) or Al (R^2^ = 0.47) in bulk subsoils (Fig. 3a–b) indicates the poor association of SOC with total Al and Fe in these soils [17]. The positive relationship between TOC and LM (R^2^ = 0.68) in the forest topsoil (Fig. 4a) was expected as the SOM maintains the stability of larger soil aggregates (>250 μm) [64,65]. The negative (R^2^ = 0.58) relationship between TOC and SM in the topsoil (Fig. 4b) may again be indicating the importance of the different organic matter fractions and structures involved in aggregate formation and stabilisation [30,68] while the positive (R^2^ = 0.57) relationship between TOC and M in the subsoil (Fig. 4c) may possibly be a result of C translocation to lower depth [31,70].

Similar to the bulk soil trend, the negative relationship between TOC and Al in the LM (R^2^ = 0.50) in the forest topsoil (Fig. 5a) and in the SC fraction (R^2^ = 0.61) in the sugarcane subsoil (Fig. 6b) again suggests that the protection of TOC in aggregates is not explained by total Al and Fe [18,54]. Similar results were reported by Oades and Waters [62], Dalal and Bridge [71] and Zhang and Horn [26] in Alfisols, Entisols and Ultisols, respectively. However, Malepfane et al. [41] reported positive correlations between TOC and Mehlich 3 extractable Fe and Al, and concluded that this fraction of Al and Fe contributes to the stabilisation of SOC in humic soils that included the ones used in this study. The contradiction in the findings could be explained by the differences in the forms Al and Fe studied, as the current study also included those in crystalline form, which may be less active than the fractions that are soluble and those in amorphous oxides, which Malepfane et al. [41] focussed on. The stabilisation of TOC by Fe and Al in humic soils may, therefore, depend on the form in which these elements occur.

The positive relationship between Al and Fe in the M fraction in the subsoil (Fig. 5b) and the SC fraction under forest (Fig. 5c) as well as in the M fraction under sugarcane (Fig. 6a) indicates the enrichment of M and SC fractions with these elements [67]. These findings are consistent with a number of studies that have found positive correlations between these elements in a wide range of soils [5,17,23,26,50,66]. According to Dalal and Bridge [71] and Shepherd et al. [72], the M and SC fractions are formed with either Al and Fe or phyllosilicate clays serving as their nucleus. Oades and Waters [62] and Alekseeva [73] call attention to the fact that M and SC fractions seem to be stabilized mostly by short-range van-der-Waals forces and electrostatic binding largely involving Al and Fe.

## 5 Conclusions

The findings of this study show that sugarcane cultivation decreases aggregate stability, and TOC in macro-aggregates, and increases Al and Fe in all aggregates, and thus adoption of practices inclined to improve or maintain TOC as well as liming to increase pH are necessary management practices for sustainable production. The negative relationship between Al and TOC in LM of the topsoil under forest and in the SC fraction of the subsoil under sugarcane indicated that total Al and Fe do not explain the protection of TOC in aggregates in humic soils. Further work needs to be carried out using extractions designed specifically to estimate the types of Al and Fe oxides because the stabilisation of TOC by Fe and Al in humic soils may depend on the form in which these elements occur. Such studies would help provide a clearer understanding on the specific Al and Fe fractions that are important in order to develop TOC sequestration strategies that may help to mitigate any TOC losses following cultivation.

## Author contribution statement

Vusumuzi Erick Mbanjwa: Conceived and designed the experiments; Performed the experiments; Analyzed and interpreted the data; Wrote the paper.

Pardon Muchaonyerwa: Conceived and designed the experiments; Contributed on the analysis strategy; Wrote the paper

Jeffrey Charles Hughes: Conceived and designed the experiments; Wrote the paper

## Funding statement

This work was supported by the National Research Foundation (GUN 93593).

## Data availability statement

Data included in article/supplementary material/referenced in article.

## Declaration of interests statement

The authors declare no conflict of interest.

## Additional information

No additional information is available for this paper.

## References

[1] Feller C., Beare M.H. (1997). Physical control of soil organic matter dynamics in the tropics. Geoderma.

[2] Amezketa E. (1999). Soil aggregate stability: a review. J. Sustain. Agric..

[3] Six J., Elliott E.T., Paustian K. (2000). Soil macro-aggregate and micro-aggregate formation: a mechanism for C-sequestration under no tillage agriculture. Soil Biol. Biochem..

[4] Bronick C.J., Lal R. (2005). Soil structure and management: a review. Geoderma.

[5] Peng X., Yan X., Zhou H., Zhang Y.Z., Sun H. (2015). Assessing the contributions of sesquioxides and soil organic matter to aggregation in an Ultisols under long-term fertilization. Soil Tillage Res..

[6] Wang J.G., Yang W., Yua B., Yang W., Li Z.X., Cai C.F. (2016). Estimating the influence of related soil properties on macro- and micro-aggregate stability in ultisols of south-central China. Catena.

[7] Krull E.S., Baldock J.A., Skjemstad J.O. (2013).

[8] Six J., Paustian K., Elliott E.T., Combrink C. (2000). Soil structure and soil organic matter: I. Distribution of aggregate-size classes and aggregate associated carbon. Soil Sci. Soc. Am. J..

[9] Lal R. (2005). Forest soils and carbon sequestration. For. Ecol. Man..

[10] Chivenge P., Murwira H., Giller K., Mapfumo P., Six J. (2007). Long-term impact of reduced tillage and residue management on soil carbon stabilization: implications for conservation agriculture on contrasting soils. Soil Tillage Res..

[11] Chaplot V., Bouahom B., Valentin C. (2010). Soil organic carbon stocks in Laos: spatial variations, variations and controlling factors. Global Change Biol..

[12] Rabbi S.M.F., Wilson B.R., Lockwood P.V., Daniel H., Young I.M. (2014). Soil organic carbon mineralization rates in aggregates under contrasting land uses. Geoderma.

[13] Wu X., Wei Y., Wang J., Wang S., Wang J., Cai C. (2017). Eects of soil physicochemical properties on aggregate stability along a weathering gradient. Catena.

[14] Grohmann F. (1960). Distribucao de tamanho de poros entres tipos de solos do Estado ae Sao Paulo. Bragantia.

[15] Tisdall J.M., Oades J.M. (1982). Organic matter and water stable aggregates in soils. J. Soil Sci..

[16] Beare M.H., Hendrix P.F., Coleman D.C. (1994). Water-stable aggregates and organic matter fractions in conventional and no-tillage soils. Soil Sci. Soc. Am. J..

[17] Igwe C.A., Akamigbo F.O.R., Mbagwu J.S.C. (1995). Physical properties of soils of south-eastern Nigeria and the role of some aggregating agents in their stability. Soil Sci..

[18] Igwe C.A., Akamigbo F.O.R., Mbagwu J.S.C. (1999). Chemical and mineralogical properties of soils in south-eastern Nigeria in relation to aggregate stability. Geoderma.

[19] Dominy C., Haynes R. (2002). Influence of agricultural land management on organic matter content, microbial activity and aggregate stability in the profiles of two Oxisols. Biol. Fertil. Soils.

[20] Pulleman M.M., Marinissen J.C.Y. (2004). Physical protection of mineralizable C in aggregates from long-term pasture and arable soil. Geoderma.

[21] Denef K., Six J. (2005). Clay mineralogy determines the importance of biological versus a biotic process for macro-aggregates formation and stabilization. Eur. J. Soil Sci..

[22] Igwe C.A., Zarei M., Stahr K. (2005). Mineral and elemental distribution in soils formed on the river Niger floodplain, Eastern Nigeria. Aust. J. Soil Res..

[23] Kaiser K., Guggenberger G. (2007). Sorption stabilization of organic matter by microporous goethite: sorption into small pores vs. surface complexation. Eur. J. Soil Sci..

[24] Six J., Paustian K. (2014). Aggregate-associated soil organic matter as an ecosystem property and a measurement tool. Soil Biol. Biochem..

[25] Six J., Bossuyt H., Degryze S., Denef K. (2004). A history of research on the link between(micro) aggregates, soil biota, and soil organic matter dynamics. Soil Tillage Res..

[26] Zhang B., Horn R. (2001). Mechanisms of aggregate stabilization in ultisols from subtropical China. Geoderma.

[27] Barthès B.G., Kouakoua E., Larré-Larrouy M.C., Tantely M.R., Edgar F. de Luca, Azontonde A., Carmen S.V., Neves J., de Freitas Pedro L., Christian L.F. (2008). Texture and sesquioxides effects on water-stable aggregates and organic matter in some tropical soils. Geoderma.

[28] Torres-Sallan G., Rachel E.C., Lanigana G.J., Reidy B., Kenneth A.B. (2018). Effects of soil type and depth on carbon distribution within soil macro-aggregates from temperate grassland systems. Geoderma.

[29] Torres-Sallan G., Schulte R.P.O., Lanigan G.J., Byrne K.A., Reidy B., Simó I., Six J. (2017). Clay illuviation provides a long-term sink for C sequestration in subsoils. Sci. Rep..

[30] Jobbágy E.G., Jackson R.B. (2000). The vertical distribution of soil organic carbon and its relation to climate and vegetation. Ecol. Appl..

[31] Olson K.R., Al-Kaisi M.A. (2015). The importance of soil sampling depth for accurate account, account of soil organic carbon sequestration, storage, retention and loss. Catena.

[32] Wiesmeier M., Urbanskia L., Hobleya E., Langc B., von Lützowa M., Marin-Spiottad E., van Wesemaele B., Rabotf E., Ließ M., Garcia-Francoa N., Wollschlägerf U., Vogelf H.J., Kögel-Knabnera I. (2019). Soil organic carbon storage as a key function of soils - a review of drivers and indicators at various scales. Geoderma.

[33] Soil Classification Working Group (2018).

[34] Fey M. (2010).

[35] Le Bissonnais Y. (1996). Aggregate stability and assessment of soil crustability and erodibility: I. Theory and methodology. Eur. J. Soil Sci..

[36] Van Antwerpen R., Meyer J.H. (1996). Soil degradation under cultivation in northern KwaZulu-natal. Proc. South Afr. Sugar Technol. Ass..

[37] Nxumalo B.N.G. (2015).

[38] Iuss Working Group Wrb (2014). World Soil Resources Reports No. 106.

[39] McBride E.F. (1963). A classification of common sandstones. J. Sediment. Petrol..

[40] Marshall C.G.A., von Brunn V. (1999). The stratigraphy and origin of the Natal Group. S. Afr. J. Geol..

[41] Malepfane M.N., Muchaonyerwa P., Hughes J.C., Zengeni R. (2022). Land use and site effects on the distribution of carbon in some humic soil profiles of KwaZulu-Natal, South Africa. Heliyon.

[42] Yost L.J., Hartemink A.E. (2019). Soil organic carbon in sandy soils: a review. Adv. Agron..

[43] Bouyoucos G.J. (1962). Hydrometer method improved for making particle size analyses of soils 1. J. Agron..

[44] Hillier S. (2003). Quantitative analysis of clay and other minerals in sandstones by X-ray powder diffraction (XRPD). Int. Assoc. Sediment. Spec. Publ..

[45] Elliott E. (1986). Aggregate structure and carbon, nitrogen, and phosphorus in native and cultivated soils. Soil Sci. Soc. Am. J..

[46] Payne R.W., Murray D.A., Harding S.A., Baird D.B. (2011).

[47] Du Toit B. (1993).

[48] Hartemink A.E. (1998). Changes in soil fertility and leaf nutrient concentration at a sugar cane plantation in Papua New Guinea. Commun. Soil Sci. Plant Anal..

[49] Fey M.V., Manson A.D., Schutter R. (1990). Acidification of the pedosphere. South Afr. J. Sci..

[50] Jim C.Y. (2003). Conservation of soils in culturally protected woodlands in rural Hong Kong. For. Ecol. Man..

[51] Gubevu S.J. (1997).

[52] Guo L.B., Gifford R.M. (2002). Soil carbon stocks and land use change: a meta-analysis. Global Change Biol..

[53] Blanco-Canqui H., Lal R. (2004). Mechanism of carbon sequestration in soil aggregates. Crit. Rev. Plant Sci..

[54] Zhao L., Cai C.F., Zhi-Hua S., Tian-Wei W. (2005). Aggregate stability and its relationship with some chemical properties of red soils in subtropical China. Pedosphere.

[55] Zhao J., Chena S., Hua R., Li Y. (2017). Aggregate stability and size distribution of red soils under different land uses integrally regulated by soil organic matter, and iron and aluminum oxides. Soil Tillage Res..

[56] Balabane M., Balesdent J. (1996). Major contribution of roots to soil carbon storage inferred from maize cultivated soils. Soil Biol. Biochem..

[57] Howard P.J.A., Howard D.M. (1990). Use of organic carbon and loss-on-ignition to estimate soil organic matter in different soil types and horizons. Biol. Fertil. Soils.

[58] Klein C., Dutrow B. (2007).

[59] Yost L.J., Roden E.E., Hartemink A.E. (2019). Geochemical fingerprint and soil carbon of sandy Alfisols. Soil Syst..

[60] Le Bissonnais Y., Duval O., Gaillard H. (2002). Measurement of aggregate stability for the evaluation of the sensitivity to sealing and crusting. INRA Orlèans Unitè de Science du Sol..

[61] Blair N. (2000). The impact of cultivation and sugarcane trash management on soil carbon fractions and aggregate stability. Soil Tillage Res..

[62] Oades J.M., Waters A.G. (1991). Aggregate hierarchy in soils. Aust. J. Soil Res..

[63] Wakindiki I.I.C., Ben-Hur M. (2002). Soil mineralogy and texture effects on crust micromorphology, infiltration and erosion. Soil Sci. Soc. Am. J..

[64] Roth C.H., Castro Filho C., de Mediros G.B. (1991). Análise de fatores f'ısicos e qu'ımicos relacionados com a agregação de um Latossolo Roxo distrófico. R. Bras. Ci. Solo.

[65] Paustian K., Collins H.P., Paul E.A., Paul E.A., Paustian K., Elliot E.T., Cole C.V. (1997). Soil Organic Matter in Temperate Agroecosystems.

[66] Schmidt M.W.I., Torn M.S., Abiven S., Dittmar T., Guggenberger G., Janssens I.A., Kleber M., Kogel-Knabner I., Lehmann J., Manning D.A.C., Nannipieri P., Rasse D.P., Weiner S., Trumbore S.E. (2011). Persistence of soil organic matter as an ecosystem property. Nature.

[67] Castro Filho C., Lourenço A., Guimarãesde M.F., Fonseca I.C.B. (2002). Aggregate stability under different management systems in a red Latosol in the State of Paraná, Brasil. Soil Tillage Res..

[68] Totsche K.U., Amelung W., Gerzabek M.H., Guggenberger G., Klumpp E., Knief C., Lehndorff E., Mikutta R., Peth S., Prechtel A., Ray N. (2019). Kö gel-Knabner, I., 2018. Micro-aggregates in soils. J. Plant Nutr. Soil Sci..

[69] Zhang S., Li Q., Zhang X., Wei K., Chen L., Liang W. (2012). Effects of conservation tillage on soil aggregation and aggregate binding agents in black soil of Northeast China. Soil Tillage Res..

[70] Lugato E., Simonetti G., Morari F., Nardi S., Berti A., Giardini L. (2010). Distribution of organic and humic carbon in wet-sieved aggregates of different soils under long-term fertilization experiment. Geoderma.

[71] Dalal R.C., Bridge B.J., Carter M.R., Stewart B.A. (1996). Structure and Organic Matter Storage in Agricultural Soils.

[72] Shepherd T.G., Saggar S., Newman R.H., Ross C.W., Dando J.L. (2001). Tillage induced changes to soil structure and organic carbon fractions in New Zealand soils. Aust. J. Soil Res..

[73] Alekseeva T.V. (2007). Soil microstructure and factors of its formation. Eur. J. Soil Sci..

